# Measurement of Flexion Angle of the Finger Joint during Cylinder Gripping Using a Three-Dimensional Bone Model Built by X-Ray Computed Tomography

**DOI:** 10.1155/2019/2839648

**Published:** 2019-01-06

**Authors:** Satoshi Shimawaki, Takuma Murai, Masataka Nakabayashi, Hideharu Sugimoto

**Affiliations:** ^1^Department of Mechanical and Intelligent Engineering, Utsunomiya University, 7-1-2 Yoto, Utsunomiya, 321-8585 Tochigi, Japan; ^2^Department of Radiology, School of Medicine, Jichi Medical University and Hospital, 3311-1 Yakushiji, Shimotsuke, 329-0498 Tochigi, Japan

## Abstract

Motion analysis of the thumb and the four fingers during human gripping of a cylindrical object is a prerequisite for designing motion mechanisms in electronic arm prostheses and robotic hands. Conventional measurement methods include the use of angle sensors or multiple video recording of markers. In the present study, we performed X-ray computed tomography (CT) imaging on fingers gripping cylinders of three different diameters (10, 60, and 120 mm) and constructed a bone model based on these CT images to directly measure the flexion angle of each finger joint. We then compared the results with the flexion angles of joints measured using other methods. The subjects comprised 10 Japanese men with no hand injuries or diseases. Our results showed that smaller cylinder diameters were associated with significant increases in the flexion angle of all the joints of the four fingers. When focusing on the distal interphalangeal joint (DIP), there was no significant difference between any of the fingers for each of the cylinders, except between the index and middle fingers for the 10 mm-diameter cylinder. When focusing on the 10 mm-diameter cylinder, the flexion angle of the proximal interphalangeal joint (PIP) of each finger was significantly larger than that of the DIP and metacarpophalangeal joint (MP). However, no such significant difference was noted for the 120 mm-diameter cylinder. The coupling ratio (CR), which is the ratio of the flexion angles of the DIP and PIP, was significantly smaller for the 10 mm-diameter cylinder than for the 60 mm-diameter cylinder. However, there were no significant differences in CR between any of the fingers. A comparison of our study results with those derived using other methods indicated quantitative consistency for the DIP and PIP. However, for the MP, we noted differences that may be explained by the difficulty in determining the longitudinal axis of the metacarpal using other methods.

## 1. Introduction

Several studies have investigated the gripping of an object by human fingers. Napier [1] classified gripping patterns into power and precision grips based on the anatomical and functional viewpoints. Power grip involves the manipulation of an object using the hand and arm, whereas precision grip involves the manipulation of an object using the fingers. Landsmeer [2] also classified gripping into two types, namely power grip and precision handling. With the former, the finger pulp is in an opposing position to the palm, whereas with the latter, the finger pulp is in an opposing position to the thumb pulp. With the power grip, the fingers and palm pressed against the object make it possible to convey a strong force to the object. The main type of gripping employed when holding a cylinder is the power grip.

When designing motion mechanisms for electronic arm prostheses and humanoid fingers (robotic hands), it is important to evaluate motion analysis of the thumb and the four fingers when a person grips a cylindrical object. Moreover, human motion data are crucial in enabling such devices to perform human-like finger movements. The use of an actuator for each joint would result in a bulky and intricate electronic arm prosthesis. Therefore, coordinated coupling between joints should be considered to reduce the number of actuators [3, 4]. Such data could also be useful for hand and finger function diagnosis [5].

Studies have used different methods to measure the joint angles of the thumb and the four fingers while gripping a cylinder. Some of these methods employed an angle sensor attached to the back of the hand [6–8], whereas others used multiple video cameras recording the markers affixed to the back of the hand [9–13]. Although a high-precision angle measurement can be performed, the angles measured can be indirectly affected by the skin and subcutaneous tissue because the markers or angle sensors are affixed to the skin surface. To avoid this bias, we applied a method based on a bone model that was generated for numerical simulation based on X-ray CT images from our previous study [14] to measure the joint angle. This method can measure the joint angle more directly.

In this study, we performed X-ray CT imaging on the hands gripping cylinders with three different diameters and constructed bone models for the distal phalanx, middle phalanx, and proximal phalanx and for the second to fifth metacarpal of the four fingers excluding the thumb. We measured the flexion angle of each joint and compared the results with the flexion angles measured by other researchers using other methods.

## 2. Methods

### 2.1. Subjects

The subjects comprised 10 Japanese men with no hand injuries or diseases, with ages ranging from 21 to 25 years, mean height of 173.0 ± 4.4 cm (mean ± standard deviation), mean weight of 65.0 ± 10.1 kg, mean body fat percentage of 16.7 ± 5.2%, and mean body mass index of 21.7 ± 3.1. The mean hand length of subjects (distance from the tip of the middle finger to the wrist crease) was 185.8 ± 8.1 mm. The mean palm length (distance from the palmophalangeal crease of the middle finger to the wrist crease) was 107.7 ± 5.8 mm, and the mean hand width (distance from the radial surface of the MP of the index finger to the medial surface of the distal palm crease) was 86.3 ± 5.9 mm. This study was approved by the Institutional Review Board for Research on Human Subjects of Utsunomiya University (Approval no. H17-0016). Before performing the experiment, subjects were given thorough explanations of the purpose and details of the study, and each subject provided written consent.

### 2.2. CT Imaging and Three-Dimensional Bone Model Construction

**Figure 1:**
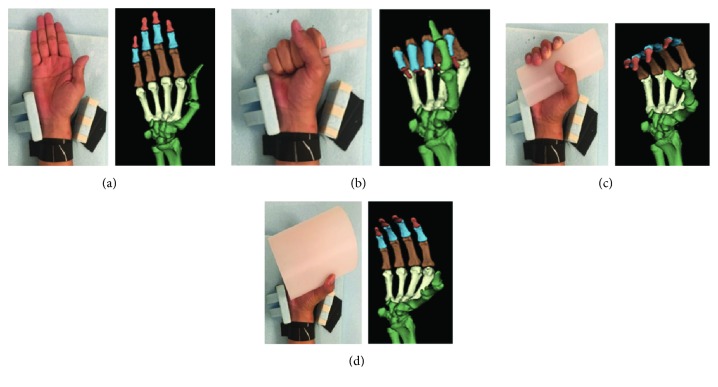
CT imaging positions and three-dimensional bone models. (a) Basic position, (b) gripping a 10 mm-diameter cylinder, (c) gripping a 60 mm-diameter cylinder, and (d) gripping a 120 mm-diameter cylinder.

Cross-sectional images distal from the midshaft of the forearm (spatial resolution: 512 × 512) were obtained with a field of view of 180 mm and slice thickness of 0.5 mm using an X-ray CT scanner (SOMATOM Definition AS; Siemens AG, Munich, Germany). The X-ray tube voltage was 120 kV and the tube current was 48 mA. CT images were obtained with the hands in the following four positions: basic position not gripping any cylinder and with all fingers extended, gripping a 10 mm-diameter cylinder, gripping a 60 mm-diameter cylinder, and gripping a 120 mm-diameter cylinder (Figure 1). All cylinders were made of polypropylene. The gripping type was power grip using the five fingers including the thumb and the palm surface. However, the thumb was set opposing the other fingers. During CT imaging, the distal and central parts of the forearm and distal part of the upper arm were fixated with bands so that only the five fingers and palm could move. Subjects were instructed to hold their wrist in a neutral position. Radioulnar deviation of the wrist was restricted using holders fixed to the jig, and flexion of the wrist was restricted by contacting the back of the hand with the jig.

Open source software (3D Slicer; ver. 4.5) for visualizing medical images was used to construct the three-dimensional bone models based on CT images of all bones distal from the distal portions of the radial and ulnar visually (Figure 1). Coordinates were set in the three-dimensional bone models. The origin of the coordinate system was set as the concave part of the dorsal surface of the capitate in the basic position. The *y*-axis was a line passing through the origin and the direction of the long axis of the third metacarpal (proximal direction was set as positive), the *x*-axis was a line passing through the origin and running in the palmardorsal direction to intersect the *y*-axis (palm direction was set as positive), and the *z*-axis was a line passing through the origin and running in the radioulnar direction (radial direction was set as positive) to intersect the *y*-axis [15].

### 2.3. Flexion Angles for the Interphalangeal Joints

**Figure 2:**
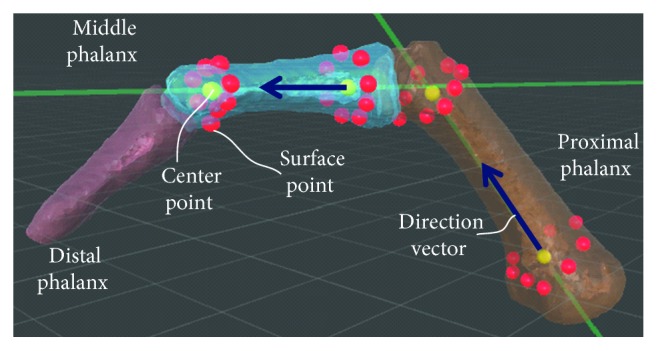
Method for calculating the flexion angle of the PIP. Direction vectors from the center of the proximal base to the center of the distal head of the middle phalanx and proximal phalanx were calculated, and the angle created by these two direction vectors was considered to be the flexion angle of the PIP.

Bone models for each cylinder gripped were used to measure the flexion angles of the distal interphalangeal joints (DIPs), proximal interphalangeal joints (PIPs), and metacarpophalangeal joints (MPs) of the four fingers (index, middle, ring, and little). The thumb was excluded from the measurements because the location of the thumb varies greatly among individuals when gripping a cylindrical object. For example, we used the following method to calculate the flexion angle of the PIP of the index finger (Figure 2). Eight surface points with intervals of approximately 45° are selected from the outer circumference of the distal head of the middle phalanx of the index finger. The coordinate of the center of the distal head of the middle phalanx is determined on the basis of the coordinates of these eight points. The coordinates of the centers of the proximal base of the middle phalanx, the distal head, and the proximal base of the proximal phalanx are determined in a similar manner. For both bones, direction vectors from the center of the proximal base to the center of the distal head are calculated and the angle created by these direction vectors is considered the flexion angle for the PIP of the index finger. The flexion angles for the DIPs, PIPs, and MPs of the four other fingers are similarly calculated.

When gripping an object, both the DIP and PIP are flexed. However, it has been reported that the flexion angle proportions of these joints are independent of the size of the object [6]. Therefore, we defined the flexion angle proportions of the DIP and PIP as the coupling ratio (CR).

### 2.4. Statistical Analysis

A three-way ANOVA with repeated measurement (diameter *A* (3) × finger *B* (4) × joint *C* (3)) was conducted for the flexion angle as the dependent variable (SPSS Statistics, Ver. 22, IBM). Sphericity was confirmed using Mauchly's sphericity test. If the sphericity hypothesis was denied, then the Greenhouse–Geisser correction was used. Three-way ANOVA results indicated that secondary (*A* × *B* × *C*) and primary (*A* × *B*, *A* × *C*, and *B* × *C*) interactions were significant (Table 1). We performed simple main effect test as well as multiple comparisons using the Bonferroni method.

A two-way ANOVA with repeated measurement (diameter *A* (3) × finger *B* (4)) was conducted for CR as the dependent variable. Sphericity was confirmed using Mauchly's sphericity test. If the sphericity hypothesis was denied, then the Greenhouse–Geisser correction was used. The results of the two-way ANOVA indicated that interaction (*A* × *B*) was not significant (*F*(6, 54) = 0.505, n.s.), but the main effect (*A*, *B*) was significant (*A*: *F*(2, 18) = 4.981, *P* = 0.019; *B*: *F*(2.247, 20.22) = 3.436, *P* = 0.047). Therefore, a multiple comparison was conducted using the Bonferroni method.

## 3. Results

### 3.1. Joint Flexion Angles

Table 2 shows the mean flexion angle and standard deviation in each joint (DIP, PIP, and MP) from the index finger to the little finger when cylinders of the three different diameters (10, 60, 120 mm) were gripped. Two of the three factors were selected. Multiple comparisons of the remaining factor were analyzed at combinations of all levels in the two selected factors using the Bonferroni method. The level of significance was set at *α* = 0.05.

#### 3.1.1. “Diameter *A*” Factor

Table 3 shows results for multiple comparisons of the “Diameter *A*” factor for combinations of all levels in the “Finger *B*” and “Joint *C*” factors. Although no significant differences were noted for the index finger in some comparisons, the flexion angle was found to significantly increase with smaller cylinder diameters. The result shows that all joints in all fingers have larger flexion angles when gripping an object with a small cylinder diameter compared with when gripping an object with a larger cylinder diameter.

#### 3.1.2. “Finger *B*” Factor

Table 4 shows the results for multiple comparisons of the “Finger *B*” factor for combinations of all levels in the “Diameter *A*” and “Joint *C*” factors. When focusing on the DIP in particular, the only significant difference noted between any of the levels was between the index finger and the middle finger for the 10 mm-diameter cylinder. The result indicates that DIP joints in all fingers contribute equally when gripping a cylindrical object. When focusing on the PIP in particular, the flexion angle of the little finger was significantly smaller than that of the other fingers in conditions for which significant differences were observed. The result shows that the DIP joint in the little finger contributes less than that in other fingers when gripping a cylindrical object. In addition, the DIP joints in all the other fingers were found to contribute equally. When focusing on the MP in particular, although the flexion angle of the index finger for the 10 mm-diameter cylinder was significantly smaller than that of the middle finger, the flexion angle of the index finger for the 120 mm-diameter cylinder was significantly larger than the flexion angle of the other fingers. This result shows that the contribution of a finger changes according to the diameter of an object.

#### 3.1.3. “Joint *C*” Factor

Table 5 shows the results for multiple comparisons of the “Joint *C*” factor for combinations of all levels in the “Diameter *A*” and “Finger *B*” factors. When focusing on the 10 mm-diameter cylinder in particular, the flexion angle of the PIP of each finger was significantly larger than that of the DIP and MP. However, no significant difference was noted for the 120 mm-diameter cylinder. When focusing on the 90 mm-diameter cylinder, although the flexion angle of the DIP of the middle and ring fingers was significantly smaller than that of the PIP and MP, no significant differences were noted between any joint for the index and little fingers. This result indicates that the PIP joints in all fingers contribute the most when the diameter of the gripped object is small. However, the contribution of all fingers becomes more equal with an increase in the diameter of the gripped object.

### 3.2. Coupling Ratio (CR)

Table 6 shows the CR, which is the ratio of the flexion angles of the DIP and PIP. Results of multiple comparisons of main effects using the Bonferroni method indicated that for “Diameter *A*” and “Finger *B*” factors, the CR of the 10 mm-diameter cylinder was significantly smaller than that of the 60 mm-diameter cylinder. No significant differences were noted among any of the levels in the “Finger *B*” factor. CR hardly changed when the cylinder diameter was large. In other words, the DIP and PIP joints in all fingers were found to flex synchronously under such conditions.

## 4. Discussion

Several studies have measured the flexion range of motion (ROM) of each finger joint [16–18]. The mean flexion ROM of the DIP, PIP, and MP was 68°, 104°, and 80° for the index finger; 70°, 107°, and 85° for the middle finger; 66°, 107°, and 87° for the ring finger; and 69°, 104°, and 86° for the little finger, respectively [18]. The flexion angles of the PIPs when holding a 10 mm-diameter cylinder have almost the same mean flexion ROM, as shown in Table 2. However, the flexion angles of the DIP of the index finger and the MP of the little finger when gripping the 10 mm-diameter cylinder in our study were smaller than the mean flexion ROM.

Several studies have also measured the flexion angles of each finger when gripping a cylinder. Lee and Rim [9] simultaneously recorded multiple markers affixed to the finger dorsal surface using four video cameras and calculated the three-dimensional coordinates of the markers to measure the flexion angles of the DIP, PIP, and MP of the four fingers. However, because the range of diameters of the gripped cylinders (25–50 mm) was relatively narrow, it was concluded that although the flexion angles of the PIP and MP increase as the cylinder diameter decreases, the flexion angle of the DIP remains fixed regardless of the cylinder size. Takano et al. [10] used the same method as Lee and Rim [9] to measure the flexion angles of the five fingers, including the thumb. Cylinder diameters ranged from 18 mm to 73 mm, with a decrease in the flexion angles of the four fingers, excluding the thumb, with increases in the cylinder diameter. Gülke et al. [6] used a sensor glove with 14 joint angle sensors affixed to the back of the hand to measure the flexion angles of the five fingers. Cylinder diameters ranged from 40 mm to 120 mm. Similar to the results reported by Takano et al., the flexion angles of the four fingers, excluding the thumb, decreased with increasing cylinder diameters. Lee and Jung [11] affixed multiple reflective markers to the back of the hand and used the VICON system to measure the flexion angles of the five fingers in different gripping positions. Cylinder diameters ranged from 20 mm to 80 mm. As with the results reported by Takano et al., the flexion angles of the four fingers, excluding the thumb, decreased with increasing cylinder diameters. However, the flexion angles when a cylinder with a smaller diameter was gripped were smaller than those obtained in Takano et al.'s study [10].

**Figure 3:**
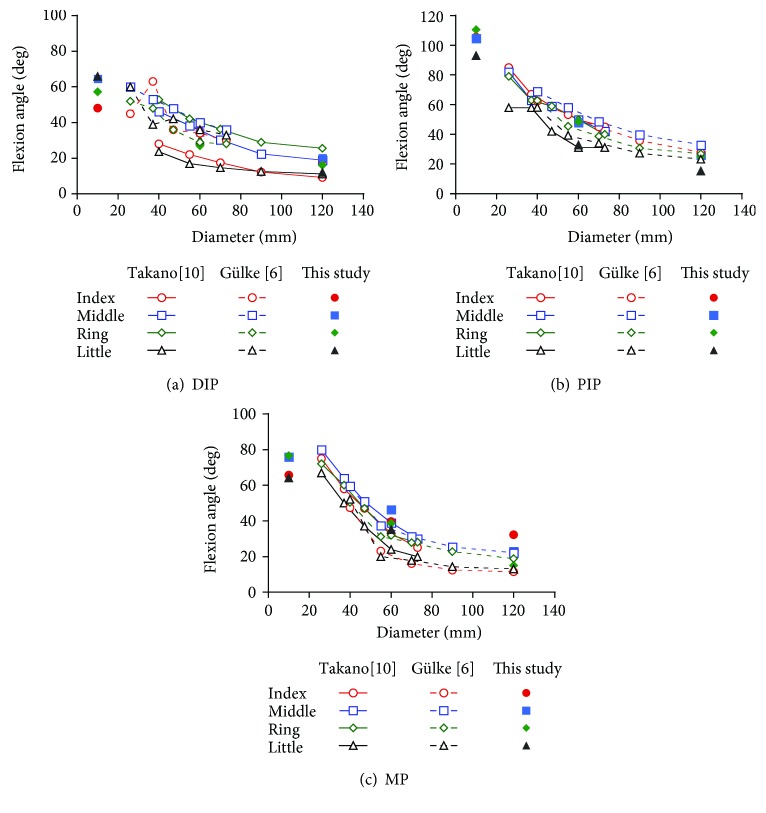
A comparison of flexion angles measured in our study with those measured in Takano et al.'s and Gülke et al.'s studies [6, 10].

Figure 3 compares flexion angles measured in our study with those measured in Takano et al.'s and Gülke et al.'s studies [6, 10]. To simplify the results, the standard deviation is not shown.  Figure 3(a) shows that when the flexion angles of the DIP are compared, similar trends are noted for the 10 mm-diameter cylinder in our study as observed by Takano et al. For the 60 mm-diameter cylinder, our results for the middle finger are very consistent with those reported by Takano et al. and Gülke et al., whereas for the other fingers, our results are consistent only with those reported by Takano et al. For the 120 mm-diameter cylinder, our results for the middle and little fingers are highly consistent with those reported by Gülke et al., whereas for the other fingers, differences were observed.  Figure 3(b) shows that when the flexion angles of the PIP were compared, similar trends were noted for the 10 mm-diameter cylinder between our study and Takano et al.'s study for all fingers, except for the little finger. For the 60 mm-diameter cylinder, results for all joints are highly consistent with those reported by Takano et al. and Gülke et al. For the 120 mm-diameter cylinder, results for the index and little fingers were different from those reported by Gülke et al. but highly consistent for the other fingers.  Figure 3(c) shows that when the flexion angles of the MP were compared, results were different from those reported by Takano et al. for the 10 mm-diameter cylinder. Differences were also noted for the 60 mm-diameter cylinder compared with those reported by Takano et al. and Gülke et al. For the 120 mm-diameter cylinder, results were highly consistent with those reported by Gülke et al., except for the index finger. A comparison with the results of other researchers who used different measurement methods indicated qualitative consistency, with the flexion angle of each joint shown to decrease with increasing cylinder diameters. While quantitative consistency was noted for the DIP and PIP, large differences were observed for the MP compared with results reported by other researchers. This may have been because other researchers measured flexion angles using video measurement of markers or angle sensors affixed to the back of the hand, and although it is relatively easy to identify long axes of the distal, middle, and proximal phalanxes, it is difficult to determine the long axis of the metacarpal.

**Figure 4:**
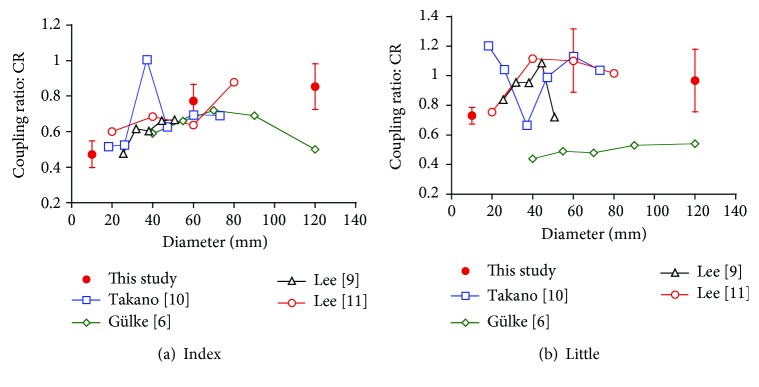
A comparison of the CR obtained in our study with those reported by other researchers. 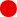: this study, 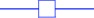: Takano et al. [10], 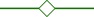: Gülke et al. [6], 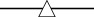: Lee and Rim [9], and 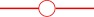: Lee and Jung [11].

Figure 4 compares the results of the CR obtained in our study with those reported by other researchers. A comparison of the results obtained for the index finger shown in Figure 4(a) indicates that the CR increases with increases in the cylinder diameter of up to 60 mm. For cylinders of ≥60 mm, the CR remains fixed or decreases. A comparison of the results of the little finger shown in Figure 4(b) indicates that the CR increases with increases in the cylinder diameter of up to 60 mm. For cylinders of ≥60 mm, the CR remains almost fixed. However, the standard error indicates large individual variations. Because the results reported by Gülke et al. indicated different trends to those reported by other researchers, some sort of measurement error may have occurred. Thus, because the CR increases and flexion angles of the DIP and PIP decrease with a cylinder diameter of up to 60 mm (Figure 3), adjustments against the diameter change are mainly conducted by the PIP. Moreover, because the CR is mainly fixed for cylinder diameters of ≥60 mm while joint angles of the DIP and PIP decrease (Figure 3), the adjustments in this case appear to be conducted by both joints.

Table 3 shows that the flexion angles of all joints of the four fingers significantly increased with a decrease in the cylinder diameter. These results, which were also observed by other researchers, indicate that all joints are mobile when the object is gripped. However, as shown in Table 6, the CR (the ratio of the flexion angles of the DIP and PIP) was different for all gripped objects. The CR was significantly smaller when a 10 mm-diameter cylinder was gripped than when a 60 mm-diameter cylinder was gripped. Therefore, the flexion angle of the PIP changes appears to be greater when the gripped object has a diameter changing from 10 mm to 30 mm. According to Table 4, flexion angles of the DIP of the four fingers were almost fixed when objects were gripped and the flexion angle of the PIP of the little finger was smaller than that of the other fingers. According to Table 5, when a 10 mm-diameter cylinder was gripped, the flexion angle of the PIP of all fingers was significantly larger than that of the other joints. However, when a 120 mm-diameter cylinder was gripped, no significant differences were noted between the flexion angle of the PIP and the flexion angles of the other joints, indicating that greater changes were observed in the flexion angles of the PIP.

Several limitations accompany the measurement of the flexion angle using X-ray CT imaging. First, this technique is a low invasive measurement because of the use of X-ray. This makes it difficult to perform measurements in several different conditions on each subject. Therefore, the radiation dose (CTDI_vol_) used in this study was fairly low (5 mGy). Second, only the flexion angle can be measured when the cylindrical object is gripped. When video imaging or a sensor glove is used, the flexion angle during prehension before gripping the cylinder can be simultaneously measured. Third, measurements could only be performed in the CT imaging room. The fourth limitation was that the flexion angles of the thumb joints were not measured. With a sensor glove, flexion angles can be measured during daily living. Thus, our data could be used to verify data measured using video imaging or sensor glove methods.

## 5. Conclusions

In the present study, we performed X-ray CT imaging on fingers gripping cylinders of three different diameters (10, 60, and 120 mm) and constructed a bone model based on these CT images to directly measure the flexion angle of each finger joint. Our results showed that smaller cylinder diameters were associated with significant increases in the flexion angle of all the joints of the four fingers. When focusing on the 10 mm-diameter cylinder, the flexion angle of the PIP of each finger was significantly larger than that of the DIP and MP. However, no significant difference was noted for the 120 mm-diameter cylinder. The coupling ratio (CR), which is the ratio of the flexion angles of the DIP and PIP, was significantly smaller for the 10 mm-diameter cylinder than for the 60 mm-diameter cylinder. However, there were no significant differences of the CR between any of the fingers.

## Figures and Tables

**Table 1:** Three-way ANOVA with repeated measurement (diameter *A* (3) × finger *B* (4) × joint *C* (3)). The dependent variable was the flexion angle.

Source of variation	Degree of freedom	Type III SS	Mean squares	*F*	*P*
Diameters *A*^a^	1.149	207,673.697	180,797.809	1025.177	<0.001
Fingers *B*^b^	3	4014.324	1338.108	27.279	<0.001
Joints *C*^c^	2	26,868.242	13,434.121	28.683	<0.001
*A* × *B*	6	2151.941	358.657	12.524	<0.001
*A* × *C*	2.301	19,652.346	8542.455	23.408	<0.001
*B* × *C*	6	2653.076	442.179	6.792	<0.001
*A* × *B* × *C*	12	2488.546	207.379	3.036	0.001

^a^Diameters—10 mm, 60 mm, and 120 mm. ^b^Fingers—index, middle, ring, and small. ^c^Joints—DIP, PIP, and MP. SS: sums of squares.

**Table 2:** Mean flexion angle of each joint from the index to the little finger when cylinders of the different diameters were gripped. Numbers in the lower brackets indicate standard deviation.

Diameter	Index (deg)			Middle (deg)		
	DIP	PIP	MP	DIP	PIP	MP

10 mm	48.2 (22.7)	105.5 (9.2)	65.6 (7.9)	64.8 (16.4)	104.8 (9.8)	75.9 (5.5)
60 mm	35.2 (6.8)	48.0 (7.7)	39.7 (11.8)	34.5 (7.5)	48.1 (8.1)	46.3 (15.4)
120 mm	18.9 (6.1)	24.2 (6.5)	32.2 (5.5)	20.0 (12.0)	25.9 (6.7)	22.9 (10.3)

Diameter	Ring (deg)			Small (deg)		
	DIP	PIP	MP	DIP	PIP	MP

10 mm	57.2 (16.8)	110.5 (6.1)	76.6 (14.9)	65.8 (8.9)	93.0 (13.3)	64.1 (12.1)
60 mm	27.1 (7.1)	48.7 (6.8)	38.7 (12.5)	30.0 (9.6)	32.8 (11.3)	35.2 (15.9)
120 mm	16.1 (6.4)	24.7 (9.1)	15.1 (8.5)	11.9 (5.3)	15.2 (5.2)	12.6 (5.7)

**Table 3:** *p* value as calculated with multiple comparisons of the “Diameter *A*” factor.

Comparison	Index			Middle		
	DIP	PIP	MP	DIP	PIP	MP

*A*1 vs *A*2	0.155 n.s.	**<0.0005** *A*1 > *A*2	**<0.0005** *A*1 > *A*2	**<0.0005** *A*1 > *A*2	**<0.0005** *A*1 > *A*2	**<0.0005** *A*1 > *A*2
*A*2 vs *A*3	**<0.0005** *A*2 > *A*3	**<0.0005** *A*2 > *A*3	0.084 n.s.	**0.001** *A*2 > *A*3	**<0.0005** *A*2 > *A*3	**<0.0005** *A*2 > *A*3
*A*1 vs *A*3	**0.011** *A*1 > *A*3	**<0.0005** *A*1 > *A*3	**<0.0005** *A*1 > *A*3	**<0.0005** *A*1 > *A*3	**<0.0005** *A*1 > *A*3	**<0.0005** *A*1 > *A*3

Comparison	Ring			Small		
	DIP	PIP	MP	DIP	PIP	MP

*A*1 vs *A*2	**0.001** *A*1 > *A*2	**<0.0005** *A*1 > *A*2	**0.001** *A*1 > *A*2	**<0.0005** *A*1 > *A*2	**<0.0005** *A*1 > *A*2	**0.001** *A*1 > *A*2
*A*2 vs *A*3	**<0.0005** *A*2 > *A*3	**<0.0005** *A*2 > *A*3	**<0.0005** *A*2 > *A*3	**<0.0005** *A*2 > *A*3	**0.004** *A*2 > *A*3	**0.002** *A*2 > *A*3
*A*1 vs *A*3	**<0.0005** *A*1 > *A*3	**<0.0005** *A*1 > *A*3	**<0.0005** *A*1 > *A*3	**<0.0005** *A*1 > *A*3	**<0.0005** *A*1 > *A*3	**<0.0005** *A*1 > *A*3

Significance level: *α* = 0.05. *A*1 = 10 mm, *A*2 = 60 mm, and *A*3 = 120 mm.

**Table 4:** *p* value as calculated with multiple comparisons of the “Finger *B*” factor.

Comparison	Diameter 10 mm			Diameter 60 mm			Diameter 120 mm		
	DIP	PIP	MP	DIP	PIP	MP	DIP	PIP	MP
*B*1 vs *B*2	**0.002** *B*1 < *B*2	1.0 n.s.	**0.011** *B*1 < *B*2	1.0 n.s.	1.0 n.s.	0.391 n.s.	1.0 n.s.	1.0 n.s.	**0.014** *B*1 > *B*2
*B*1 vs *B*3	0.551 n.s.	1.0 n.s.	0.729 n.s.	0.10 n.s.	1.0 n.s.	1.0 n.s.	1.0 n.s.	1.0 n.s.	**<0.0005** *B*1 > *B*3
*B*1 vs *B*4	0.084 n.s.	0.128 n.s.	1.0 n.s.	0.661 n.s.	**0.004** *B*1 > *B*4	0.874 n.s.	0.065 n.s.	0.162 n.s.	**<0.0005** *B*1 > *B*4
*B*2 vs *B*3	0.353 n.s.	0.704 n.s.	1.0 n.s.	0.191 n.s.	1.0 n.s.	0.302 n.s.	1.0 n.s.	1.0 n.s.	**0.018** *B*3 > *B*4
*B*2 vs *B*4	1.000 n.s.	**0.007** *B*2 > *B*4	**0.026** *B*2 > *B*4	0.65 n.s.	**0.003** *B*2 > *B*4	0.071 n.s.	0.097 n.s.	**0.025** *B*2 > *B*4	0.108 n.s.
*B*3 vs *B*4	0.861 n.s.	**0.006** *B*3 > *B*4	0.382 n.s.	1.0 n.s.	**0.001** *B*3 > *B*4	0.510 n.s.	0.705 n.s.	0.348 n.s.	1.0 n.s.

Significance level: *α* = 0.05. *B*1 = index, *B*2 = middle, *B*3 = ring, and *B*4 = small.

**Table 5:** *p* value as calculated with multiple comparisons of the “Joint *C*” factor.

Comparison	Index			Middle		
	10 mm	60 mm	120 mm	10 mm	60 mm	120 mm

*C*1 vs *C*2	**0.001** *C*1 < *C*2	0.050 n.s.	0.508 n.s.	**<0.0005** *C*1 < *C*2	**0.034** *C*1 < *C*2	0.958 n.s.
*C*1 vs *C*3	0.125 n.s.	0.636 n.s.	**<0.0005** *C*1 < *C*3	0.148 n.s.	**0.041** *C*1 < *C*3	0.840 n.s.
*C*2 vs *C*3	**<0.0005** *C*2 > *C*3	0.538 n.s.	0.057 n.s.	**<0.0005** *C*2 > *C*3	1.0 n.s.	1.0 n.s.

Comparison	Ring			Small		
	10 mm	60 mm	120 mm	10 mm	60 mm	120 mm

*C*1 vs *C*2	**<0.0005** *C*1 < *C*2	**0.001** *C*1 < *C*2	0.095 n.s.	**0.006** *C*1 < *C*2	1.0 n.s.	0.536 n.s.
*C*1 vs *C*3	0.06 n.s.	**0.042** *C*1 < *C*3	1.0 n.s.	1.0 n.s.	0.713 n.s.	1.0 n.s.
*C*2 vs *C*3	**<0.0005** *C*2 > *C*3	0.343 n.s.	0.195 n.s.	**0.008** *C*2 > *C*3	1.0 n.s.	1.0 n.s.

Significant level: *α* = 0.05. *C*1 = DIP, *C*2 = PIP, and *C*3 = MP.

**Table 6:** Coupling ratio (CR), the ratio of the flexion angles of the DIP and PIP.

Diameter	Index	Middle	Ring	Small	All
10 mm	0.47 (0.24)	0.62 (0.16)	0.52 (0.16)	0.73 (0.18)	0.58^∗^ (0.20)
60 mm	0.77 (0.29)	0.75 (0.26)	0.58 (0.21)	1.10 (0.67)	0.80^∗^ (0.43)
120 mm	0.85 (0.41)	0.96 (0.98)	0.73 (0.44)	0.97 (0.67)	0.88 (0.65)
All	0.70 (0.35)	0.78 (0.59)	0.61 (0.30)	0.93 (0.56)	0.75 (0.48)

^∗^ *p* < 0.05.

## Data Availability

The data used to support the findings of this study are included within the article. The CT data used to support the findings of this study have not been made available because of privacy protection.
